# Sustained benefit from combined plasmapheresis and allogeneic mesenchymal stem cells transplantation therapy in systemic sclerosis

**DOI:** 10.1186/s13075-017-1373-2

**Published:** 2017-07-19

**Authors:** Huayong Zhang, Jun Liang, Xiaojun Tang, Dandan Wang, Xuebing Feng, Fan Wang, Bingzhu Hua, Hong Wang, Lingyun Sun

**Affiliations:** Department of Rheumatology and Immunology, The Affiliated Drum Tower Hospital of Nanjing University Medical School, 321 Zhongshan Road, Nanjing, 210008 China

**Keywords:** Systemic sclerosis (SSc), Plasmapheresis (PE), Mesenchymal stem cells (MSCs)

## Abstract

**Background:**

Systemic sclerosis (SSc) is an autoimmune disease involving the skin and several internal organs. Most therapies available for this disease are symptomatic. Given the difficulty in treating SSc, we conducted this study to investigate the effect of combined plasmapheresis (PE) and allogeneic mesenchymal stem cells transplantation (MSCT) therapy on SSc.

**Methods:**

Fourteen patients underwent three repeated PE treatments with subsequent pulse cyclophosphamide on days 1, 3 and 5. Patients received a single MSCT (1 × 10^6^ cells/kg of body weight) on day 8. During follow up, evaluations performed included complete physical examination, serologic testing, and organ function.

**Results:**

The mean modified Rodnan skin score (MRSS) improved from 20.1 ± 3.1 to 13.8 ± 10.2 (*P* < 0.001) at 12 months of follow up. Three patients had interstitial lung disease, all had improvement of lung function and improved computed tomography (CT) images after 12 months of combined therapy. This combined treatment also significantly decreased the anti-Scl70 autoantibody titer and serum transforming growth factor-β and vascular endothelial growth factor levels during follow up.

**Conclusion:**

The results indicate that PE combined with MSCT is a feasible treatment associated with possible clinical benefit for SSc patients.

**Trial registration:**

ClinicalTrials.gov, NCT00962923. Registered on 19 August 2009.

## Background

Systemic sclerosis (SSc) is a rare chronic autoimmune disease characterized by increased synthesis and deposition of extra-cellular matrix in skin and various internal organs. Skin involvement, important in diagnosis and classification of SSc, is an almost universal feature of SSc [1]. Depending on the extent of skin fibrosis, SSc can be classified as two main subtypes, the limited cutaneous form (lcSSc) and the diffuse cutaneous form (dcSSc). The extensive degree of skin involvement coincides with future severe internal organ manifestations, poor prognosis and mortality, at least in the early phase of dcSSc [2]. Although skin disease does not directly threaten life, the thick skin can lead to psychological stress and thus worsens the quality of life.

Many inflammatory cytokines and growth factors are associated with the onset and progression of fibrosis, such as transforming growth factor (TGF)-β, endothelin-1, interleukin (IL)-17, IL-23 and tumor necrosis factor (TNF)-α [3–6]. In general, skin disease in SSc patients is treated with immunosuppressive agents such as methotrexate (MTX) or mycophenolate mofetil (MMF) [7, 8], but are not acceptable to all Chinese patients in terms of side effects and cost. There are also various novel therapies focusing on skin disease, including anti-inflammatory immunosuppressive agents such as imatinib or rituximab, and extracorporeal shock waves [9–12]. However, assessment of their effects remains inconclusive. A few studies on plasmapheresis (PE) for treatment of SSc have demonstrated improvement in the modified Rodnan Skin Score (MRSS), decreased level of cytokines, soluble adhesion molecules and immunolaboratory markers after treatment [13–15]. However, patients often received three repeated PE treatments every 2–3 months in these studies as a high frequency of PE would increase the risk of infection due to allogeneic blood transfusion. Thus, newer therapies are needed with enhanced efficacy and less toxicity in the treatment of skin disease in SSc.

Mesenchymal stem cells (MSCs) are a subset of multipotent adult somatic stem cells that have the ability to undergo self-renewal, proliferation and pluripotent differentiation. They can be obtained from different sources such as bone marrow, umbilical cord, and adipose tissue in the human body [16–19]. Besides their multi-lineage differentiation potential [20–22], MSCs also harbor immunosuppressive activities owing to their paracrine effects and interaction with different immune cells [23–26], and limited immunogenicity with low human leukocyte antigen (HLA) I and no HLA II expression [27]. These properties of MSCs have offered a new strategy in the treatment of numerous autoimmune inflammatory diseases and demonstrated promising results in safety and efficacy. To date, MSC transplantation (MSCT) has been proved in our center to be effective in the treatment of refractory systemic lupus erythematosus, rheumatoid arthritis and inflammatory bowel disease [28–31].

Recently studies have shown deficiency of MSCs in SSc patients. Compared with healthy controls, bone marrow MSCs (BM-MSCs) isolated from SSc patients display a more mature and myofibroblast-like phenotype, which re-programs these cells toward pro-angiogenic behavior [32]. MSCs from SSc patients also exhibit abnormal functional activities, such as increased expression of TGF-β and vascular endothelial growth factor (VEGF), and impairment of endothelial cell differentiation, which may play critical roles during the development of fibrosis in SSc [33, 34]. Based on these findings, allogeneic MSCT appears a promising therapy for SSc. Indeed, one animal experiment has shown that MSCT results in lower expression of fibrotic markers in both skin and lung, and decreased levels of anti-topoisomerase (anti-scl70) autoantibodies, suggesting systemic effect of MSCs [35]. Our previous pilot study in five SSc patients also showed that MSCT results in decreased anti-nuclear antibody (ANA) titer, and improvement in the MRSS and Health Assessment Questionnaire (HAQ) [36]. Thus, we aimed to evaluate the effectiveness and safety of combination therapy with PE and MSCT for treatment of SSc on the basis of their different mechanisms in treating SSc patients.

## Methods

### Patient eligibility

Fourteen patients were recruited after approval by the Ethics Committee of Drum Tower Hospital. All patients provided signed, written, informed consent. Inclusion criteria were age 18–70 years, diagnosed as dcSSc according to 1980 American College of Rheumatology and/or the LeRoy and Medsger criteria. Exclusion criteria were: (1) pregnancy and lactation period; (2) heart failure and ventricular arrhythmia; (3) human immunodeficiency virus or hepatitis C virus seropositivity; (4) serum hepatitis B virus DNA of more than 10,000 copies/ml in patients with positive hepatitis B surface antigen; (5) presence of active untreated infectious disease; (6) presence of hepatic, portal or splenic vein thrombosis on ultrasonography; (7) presence of severe comorbid diseases (e.g., severe respiratory or cardiac disease), or presence of any type of malignancy.

### MSC culturing

The MSCs were obtained from the umbilical cord (UC). The UC-derived MSCs were prepared by the Stem Cell Center of Jiangsu Province (Jiangsu Beike Bio-Technology, Taizhou, Jiangsu). Fresh UCs was obtained from informed healthy mothers in a local maternity hospital after normal deliveries. The UCs were rinsed twice in PBS consisting of penicillin and streptomycin to remove the cord blood. Then the washed cords were cut into 1-mm^2^ pieces and floated in low-glucose DMEM containing FBS (Stemcell, Vancouver, Canada). The pieces of cord were subsequently incubated at 37 °C in a humidified atmosphere consisting of 5% CO_2_ in air. Non-adherent cells were removed by washing. The medium was replaced every 3 days after the initial plating. When well-developed colonies of fibroblast-like cells appeared after 10 days, the cultures were trypsinized and passaged into a new flask for further expansion. Flow cytometric analysis confirmed the cells expressed CD106, CD105, CD90, CD71, CD44, CD29, but not CD34, CD14, CD3 or CD45. The capacity of MSCs to differentiate along adipogenic and osteogenic lineages was evaluated as previously described [25]. MSCs at passage 3 with a purity of more than 95% were used.

### PE and MSCT

Patients received three repeated PE treatments every two days on days 1, 3 and 5, with the removal and reinfusion of 800–1000 ml plasma at each time. Patients received an intravenous cyclophosphamide (CTX) regimen to inhibit B cell proliferation triggered by rapidly decreased autoantibodies and circulating immune complex. The total amount of CTX (1.0 g/m^2^ body surface area) was divided to use on the following day after each PE (0.4–0.6 g each time). On day 8, patients received single MSC infusion (1 × 10^6^ cells/kg of body weight). Patients were discharged after at least 48 hours of observation post MSCT.

### Follow up and outcome measurements

After MSCT, each patient returned for follow up at 1, 3, 6 and 12 months. At each follow-up visit, complete physical examination, serologic testing and organ function were performed. Skin thickening was measured using the MRSS score, which was performed by at least one experienced attending physician. Serum levels of anti-scl70 IgG and the changes in TGF-β, VEGF, interferon (IFN)-γ, IL-4 and IL-10 were measured by ELISA (R&D, USA).

### Statistical analysis

GraphPad Prism 5.0 software was used for statistical analyses. Results were expressed as median (range). The paired or unpaired *t* test was used for statistical comparison of variables before and after treatment by GraphPad Prism 5.0 software. A level of *P* < 0.05 was considered statistically significant.

## Results

### Demographic findings

Fourteen patients with SSc underwent allogeneic MSCT; all of those were classified as having the diffuse cutaneous subsets of the disease. Their average age was 37.4 years (range 19–67). The average disease duration was 27 months (range 6–84). Table 1 displays patients’ demographics and drug regimens received at the time of MSCT. The mean follow-up period was 15.6 ± 4.3 months (range 7–21). Twelve patients were followed up for more than 12 months.

**Table 1:** Clinical and demographic characteristics of the patients enrolled in the study

Case	Age (years)	Sex	Duration (mo)	MRSS baseline	Organ involvement	Previous treatments	Maintain treatments	Infectious AE
1	26	M	17	26		P 15 mg/d + MTX 10 mg/w	P 5 mg/d + MTX 10 mg/w	Minor respiratory tract infection
2	21	F	24	23		P10mg/d + penicillamine 0.375/d	No treatment	
3	25	M	6	19		no treatment	No treatment	
4	35	F	30	17		P 5 mg/d + penicillamine 0.375/d	No treatment	
5	28	F	84	21	ILD acral ulcers	P 20 mg/d + CTX 0.4/2 w	P 10 mg/d + CTX 0.6/2 mo	Minor respiratory tract infection
6	43	F	60	20		Penicillamine 0.375/d	No treatment	
7	19	F	36	19		P 10 mg/d + AZA100 mg/d	P 5 mg/d + AZA50 mg/d	Minor respiratory tract infection
8	56	M	40	15	ILD	P 15 mg/d CTX 0.4/2 w	P 5 mg/d CTX 0.4/mo	Minor respiratory tract infection
9	46	F	7	21		P 10 mg/d + GTW	GTW	
10	30	F	42	18		P10mg/d + MMF 0.75 BID	No treatment	Minor respiratory tract infection
11	38	F	12	20	ILD dysphagia	P 20 mg/d + CTX 0.1 QD + GTW	P10mg + CTX 0.4/2 w + GTW	
12	67	F	6	25		P 10 mg/d + AZA 100 mg/d	P 5 mg/d + AZA 50 mg/d	Diarrhea
13	53	F	6	17		P 5 mg/d + MMF 0.75 BID	P 5 mg/d	
14	36	F	6	21		No treatment	No treatment	

Duration was from the first symptom of disease to the time receiving plasmapheresis (PE) + mesenchymal stem cell transplantation (MSCT). Skin *MRSS* modified Rodnan skin score, *AE* adverse events, *ILD* interstitial lung disease, *P* prednisone, *CTX* cyclophasphomide, *MTX* methotrexate, *AZA* azathioprine, *GTW* glycosides of *Tripterygium wilfordi*, *MMF* mycophenolate mofetil, *BID* twice daily

### Modified Rodnan skin score

**Fig. 1:**
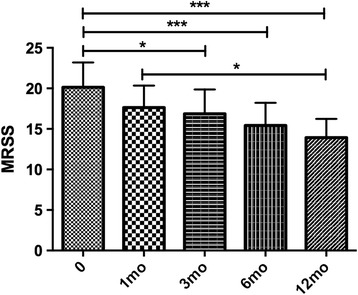
Evaluation of the modified Rodnan skin score (*MRSS*). At baseline the MRSS was 20.1 ± 3.1 (n = 14). After 1 month, the MRSS was 17.6 ± 2.7. After 3 months, the MRSS was 16.9 ± 3.0 (n = 14). After 6 months, the MRSS was 15.4 ± 2.8 (n = 14). After 12 months of treatment the mean MRSS was 13.9 ± 2.3 (n = 12). *P < 0.05,***P < 0.001

There was a significant improvement in the MRSS over the course of 1 year following the treatment. At 12 months of treatment, the mean MRSS improved from 20.1 ± 3.1 to 13.8 ± 10.2 (*P* < 0.0001) and the mean difference in MRSS was −6.2 points (95% CI −3.1 to −9.3). This change was not seen after 1 month of treatment, but was evident at 3 months, with a mean improvement of −3.3 points (−0.3 to −6.3; *P* < 0.5) and at 6 months with a mean improvement of −4.7 points (−1.7 to −7.7; *P* < 0.001) (Fig. 1).

### Non-skin fibrosis-related manifestations

**Fig. 2:**
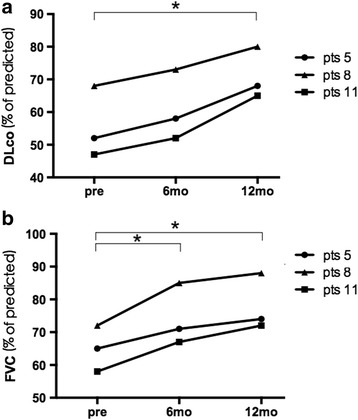
Evaluation of variables associated with interstitial lung disease (ILD) in three patents with systemic sclerosis before mesenchymal stem cell transplantation (MSCT), and at 6 months and 12 months after MSCT. **a** Diffusing capacity of the lung for carbon monoxide (*DLco*). **b** Forced vital capacity (*FVC*). *P < 0.05. *Pts* patients

**Fig. 3:**
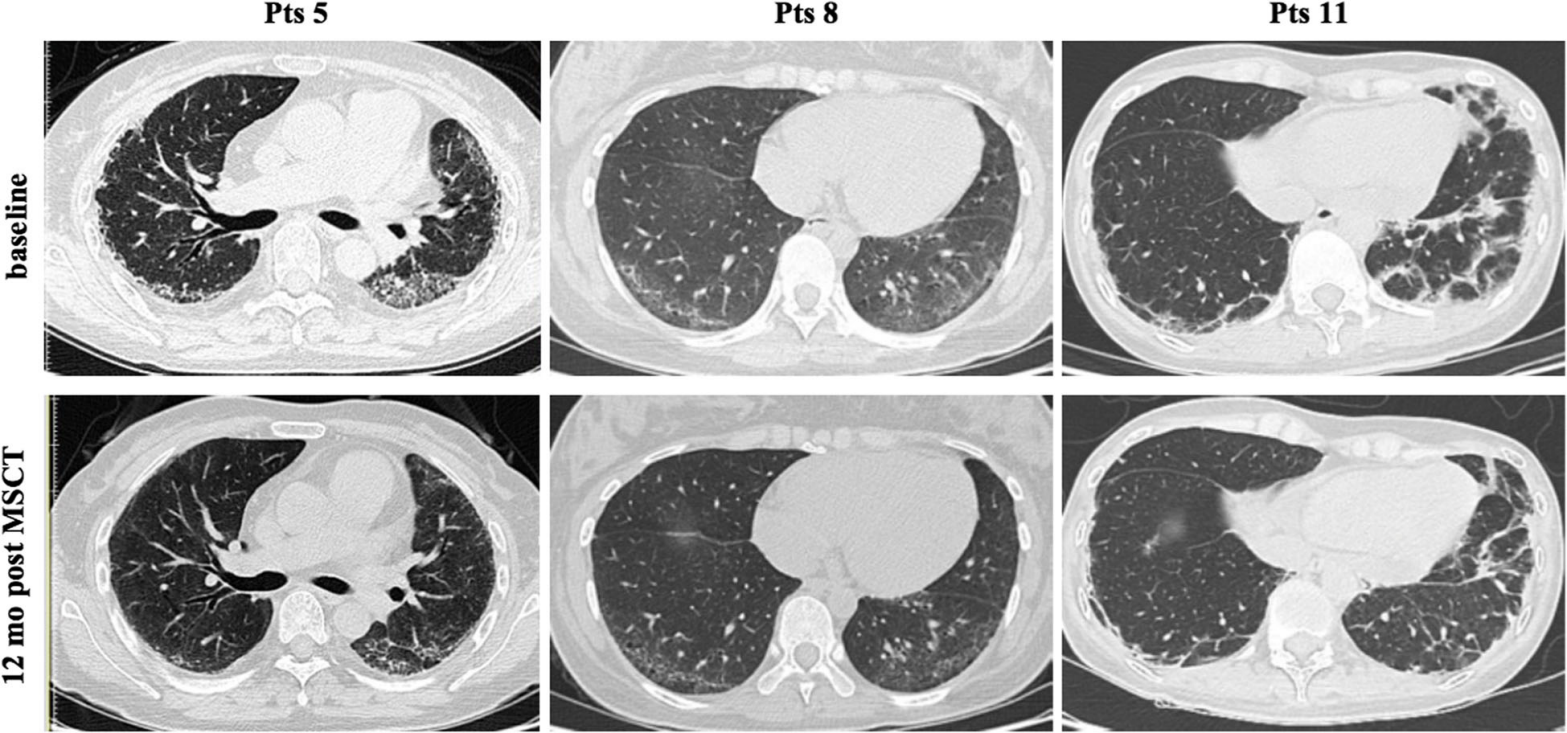
Pulmonary high-resolution computed tomography in patients with systemic sclerosis. *Upper panel* before mesenchymal stem cell transplantation (*MSCT*); *lower panel* 12 months after MSCT. *Pts* patients

Three of fourteen patients had interstitial lung disease (ILD); all patients had improvement in lung function after 12 months of combined therapy, with increased CO diffusing capacities (DLco) and forced vital capacity (FVC) (Fig. 2). Improved computed tomography (CT) images were also observed in these patients (Fig. 3). One of fourteen patients had an acral ulcer. Pain from the skin ulcer improved 1 month after combined therapy and the lesion size was reduced and healed 3 months after the treatment and did not recur till the last follow up. One of fourteen patients had dysphagia, which responded to the combined treatment during the whole follow up.

### Serology changes

**Fig. 4:**
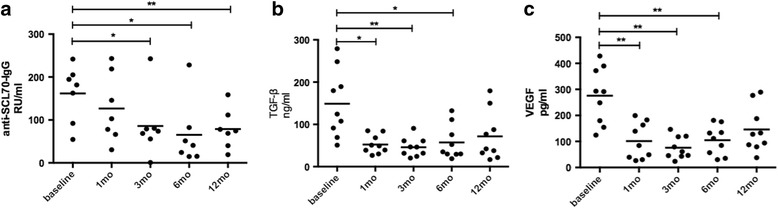
Serum anti-SCL70 IgG (**a**), transforming growth factor (*TGF*)-β (**b**) and vascular endothelial growth factor (*VEGF*) (**c**) levels in patients with systemic sclerosis were decreased after combined plasmapheresis and allogeneic mesenchymal stem cell transplantation therapy.*P < 0.05,**P < 0.01

This combined therapy significantly decreased serum anti-Scl70 autoantibody titer, TGF-β and VEGF levels (Fig. 4) during the follow up. There were no changes in the levels of IFN-γ, IL-4 or IL-10. The anti-Scl70 autoantibody titers decreased from 125.98 ± 91.13 RU/ml at baseline to 98.77 ± 88.46 RU/ml (*P* = 0.66, n = 7) at 1-month follow up; the titers were reduced to 66.91 ± 74.69 RU/ml (3-month follow up, *P* < 0.05, n = 7), 50.98 ± 71.39 RU/ml (6-month follow up, *P* < 0.05, n = 7) and 61.32 ± 52.68 RU/ml (12-month follow up, *P* < 0.01, n = 7), respectively. The serum TGF-β levels were decreased from 148.94 ± 79.85 ng/ml at baseline to 52.47 ± 21.98 ng/ml (1-month follow up, *P* < 0.05, n = 9), 45.94 ± 22.33 ng/ml (3-month follow up, *P* < 0.01, n = 9), 57.25 ± 40.56 ng/ml (6-month follow up, *P* < 0.05, n = 9) and 71.64 ± 58.20 ng/ml (12-month follow up, *P* = 0.0547, n = 9), respectively. The serum VEGF levels were decreased from 275.71 ± 108.15 pg/ml at baseline to 101.54 ± 69.88 pg/ml (1-month follow up, *P* < 0.01, n = 9), 75.84 ± 42.58 pg/ml (3-month follow up, *P* < 0.01, n = 9), 104.64 ± 56.6 pg/ml (6-month follow up, *P* < 0.01, n = 9) and 145.89 ± 88.20 pg/ml (12-month follow up, *P* = 0.1125, n = 9), respectively.

### Adverse events

No serious adverse events were observed during or immediately after PE and MSCT in any of the 14 patients. None of these patients developed graft versus host disease (GvHD) during follow up. Adverse events noted were upper respiratory tract infections reported by five patients and diarrhea reported by one patient during follow-up visits (Table 1). No serious infections occurred.

## Discussion

Skin involvement is the hallmark of SSc; improvement in skin thickening may be useful as a surrogate for improvement in survival in clinical trials [37]. Therefore, the treating skin symptoms have been the focus of investigation in many clinical trials. Recent studies have assessed different options for the treatment of skin thickness; however, most of these therapies did not show significant efficacy [38]. Herein, we proposed allogeneic MSCT combined with PE as a potential therapy for the diffuse cutaneous form of SSc. In this study, of the total of 14 patients, 11 only had diffuse sclerosis and thickening of the skin without internal organ involvement, including two newly diagnosed patients who were not receiving any treatment before and after PE + MSCT; the other 9 patients received small doses of glucocorticoid in combination with immunosuppresive agents such as MTX or MMF, etc., which 3 patients gradually stopped taking after the combined therapy, and the other 6 cases also had reduced dosage of glucocorticoids and immunosuppressants. The results indicate this combined therapy has a possible benefit in improving MRSS and reducing inflammatory markers including anti-Scl70 autoantibody titer, TGF-β and VEGF levels.

Fibrosis is the final step and is the basis of most prominent clinical manifestations in SSc patients, including skin thickness and tightness [39]. Two fundamental biological processes contribute to the development of skin fibrosis, including vasculopathy with perivascular inflammation and coagulation activation, and fibroblast activation with the excess accumulation of extracellular matrix components. Multiple factors and signaling pathways are involved in the development or persistence of skin involvement in SSc, such as TGF-β, IL-4, IL-6, platelet-derived growth factor (PDGF), IL-1, IL-13, IL-17, IL-5, monocyte chemoattractant protein (MCP)-1, VEGF and connective tissue growth factor (CTGF) [6]. Recently, some experimental studies have revealed MSC deficiency in SSc. MSCs in SSc have a different phenotype from healthy controls [32]. BM-MSCs isolated from SSc patients have upregulation of α-smooth muscle actin (SMA) and smooth muscle (SM)22α genes and reduced proliferative activity, displaying a more mature and myofibroblast-like phenotype. Cipriani P et al. have observed that BM-MSCs from SSc patients have increased senescence biomarkers, through increased activation of the IL-6 pathway [40]. Furthermore, MSCs have been proved to not only have the properties of reduced inflammatory and fibrotic processes, but also have the ability to differentiate into endothelial cells [41]. These findings supported the hypothesis that MSCs from SSc patients are structurally and functionally defective, and provide basis for allogeneic MSCT as a potential therapy for SSc patients. Our results showed that MSCT combined with PE downregulated serum levels of TGF-β, which is a major cytokine involved in early angiogenesis and latent collagen production leading to fibrosis [42]. In addition, the combined therapy was shown to reduce the levels of VEGF, which was elevated in SSc patients and could stimulate angiogenesis [6]. We also noted lower levels of anti-Scl70 antibodies after the combined treatment, suggesting reduced B cell activation.

Some studies have suggested that MSCs can exhibit a protective effect on skin tightness and thickness [36, 43]. PE is also reported to be able to improve the Rodnan skin score in small case series of SSc patients [13]. These two therapies were taken into account for combination in view of their seemingly complementary characteristics. PE could quickly remove serum pro-inflammatory substances such as inflammatory cytokines, antibodies, immunoglobulins and complements, which play major roles in the immune responses against normal skin and fibrosis. MSC could secrete anti-inflammatory cytokines, anti-fibrotic factors and trophic molecules, and differentiate into epithelial cells, which will all promote regeneration of skin. Moreover, the self-renewal capacity of MSCs would make the therapeutic effects last a long time. However, it is difficult to tell which treatment is important in the resulting clinical and laboratory test improvements. Future randomized clinical trials with larger sample sizes and long-term follow up are required to further verify the results of this study.

In addition to improving the skin lesions, the data also suggest that the combined therapy can improve internal function in these patients. ILD is a common visceral lesion in SSc patients. For more than 15 years, CTX has commonly been used in the treatment of SSc-ILD. CTX is a cytotoxic immunosuppressive agent that suppresses lymphokine production and modulates lymphocyte function. In a landmark study, the Scleroderma Lung Study Research Group noted that CTX achieved a modest but significant beneficial effect on lung function and patients’ quality of life. After 12 months of therapy, FVC increased in 49.3% of the patients who received the CTX treatment. However, unfortunately, it also provoked a serious adverse event [44]. In our study, there were three patients with different degrees of ILD, all of whom had received glucocorticoids and CTX for at least 3 ~ 6 months before the combined therapy, but without improvement in the pulmonary symptoms. However, lung function and CT images in these three patients all improved significantly, after 12 months of combined therapy, FVC increased from 65.0 ± 7.0% to 81.7 ± 8.5%. We have gradually reduced the dosage of glucocorticoids and CTX to maintaining treatment. Compared with glucocorticoids and immunosuppressive agents, this combined therapy has shown a low side reaction and good safety; only a few patients had mild upper respiratory tract infections and diarrhea.

Our study has limitations. First, we were aware of the limitation of the small sample size of this study population, thus this is an exploratory analysis. The adequacy of sample size is important to clinical trials but depends on the availability of patients. A lower prevalence of SSc explained the sample size in this trial to some extent. Second, the uncontrolled study design (automatically also not blinded) may result in a substantial overestimation of therapeutic effect.

## Conclusions

This study indicates that MSCT combined with PE is a feasible treatment associated with possible clinical benefit in SSc patients. The true value and safety will require much more robust data from a controlled trial and longer-term follow up in many more SSc patients.

## Abbreviations

ANA: Anti-nuclear antibody
BM-MSCs: Bone marrow mesenchymal stem cells
CT: Computed tomography
CTGF: Connective tissue growth factor
CTX: Cyclophosphamide
dcSSc: Diffuse cutaneous systemic sclerosis
DLco: Diffusing capacity of the lung for carbon monoxide
DMEM: Dulbecco’s modified Eagle’s medium
ELISA: Enzyme-linked immunosorbent assay
FBS: Fetal bovine serum
FVC: Forced vital capacity
GvHD: Graft versus host disease
HAQ: Health Assessment Questionnaire
HLA: Human leukocyte antigen
IL: Interleukin
ILD: Interstitial lung disease
IFN: Interferon
lcSSc: Limited cutaneous systemic sclerosis
MCP: Monocyte chemoattractant protein
MMF: Mycophenolate mofetil
MRSS: Modified Rodnan skin score
MSCs: Mesenchymal stem cells
MSCT: Mesenchymal stem cell transplantation
MTX: Methotrexate
PBS: Phosphate-buffered saline
PDGF: Platelet-derived growth factor
PE: Plasmapheresis
scl70: Topoisomerase
SM: Smooth muscle
SMA: Smooth muscle actin
SSc: Systemic sclerosis
TGF: Transforming growth factor
TNF: Tumor necrosis factor
UC: Umbilical cord
VEGF: Vascular endothelial growth factor
